# Artificial Intelligence–Powered Smartphone App to Facilitate Medication Adherence: Protocol for a Human Factors Design Study

**DOI:** 10.2196/21659

**Published:** 2020-11-09

**Authors:** Don Roosan, Jay Chok, Mazharul Karim, Anandi V Law, Andrius Baskys, Angela Hwang, Moom R Roosan

**Affiliations:** 1 Department of Pharmacy Practice and Administration College of Pharmacy Western University of Health Sciences Pomona, CA United States; 2 School of Applied Life Sciences Keck Graduate Institute Claremont Colleges Claremeont, CA United States; 3 Graduate College of Biomedical Sciences Western University of Health Sciences Pomona, CA United States; 4 Department of Pharmacy Practice School of Pharmacy Chapman University Irvine, CA United States

**Keywords:** artificial intelligence, smartphone app, patient cognition, complex medication information, medication adherence, machine learning, mobile phone

## Abstract

**Background:**

Medication Guides consisting of crucial interactions and side effects are extensive and complex. Due to the exhaustive information, patients do not retain the necessary medication information, which can result in hospitalizations and medication nonadherence. A gap exists in understanding patients’ cognition of managing complex medication information. However, advancements in technology and artificial intelligence (AI) allow us to understand patient cognitive processes to design an app to better provide important medication information to patients.

**Objective:**

Our objective is to improve the design of an innovative AI- and human factor–based interface that supports patients’ medication information comprehension that could potentially improve medication adherence.

**Methods:**

This study has three aims. Aim 1 has three phases: (1) an observational study to understand patient perception of fear and biases regarding medication information, (2) an eye-tracking study to understand the attention locus for medication information, and (3) a psychological refractory period (PRP) paradigm study to understand functionalities. Observational data will be collected, such as audio and video recordings, gaze mapping, and time from PRP. A total of 50 patients, aged 18-65 years, who started at least one new medication, for which we developed visualization information, and who have a cognitive status of 34 during cognitive screening using the TICS-M test and health literacy level will be included in this aim of the study. In Aim 2, we will iteratively design and evaluate an AI-powered medication information visualization interface as a smartphone app with the knowledge gained from each component of Aim 1. The interface will be assessed through two usability surveys. A total of 300 patients, aged 18-65 years, with diabetes, cardiovascular diseases, or mental health disorders, will be recruited for the surveys. Data from the surveys will be analyzed through exploratory factor analysis. In Aim 3, in order to test the prototype, there will be a two-arm study design. This aim will include 900 patients, aged 18-65 years, with internet access, without any cognitive impairment, and with at least two medications. Patients will be sequentially randomized. Three surveys will be used to assess the primary outcome of medication information comprehension and the secondary outcome of medication adherence at 12 weeks.

**Results:**

Preliminary data collection will be conducted in 2021, and results are expected to be published in 2022.

**Conclusions:**

This study will lead the future of AI-based, innovative, digital interface design and aid in improving medication comprehension, which may improve medication adherence. The results from this study will also open up future research opportunities in understanding how patients manage complex medication information and will inform the format and design for innovative, AI-powered digital interfaces for Medication Guides.

**International Registered Report Identifier (IRRID):**

PRR1-10.2196/21659

## Introduction

### Background

For decades, the US Food and Drug Administration (FDA) and drug manufacturers provided detailed Medication Guides to the public to effectively communicate crucial patient safety [1]. The guides include warnings, precautions, and other lists of adverse drug reactions [2]. Unfortunately, the information presented in these Medication Guides often contains complicated medical jargon. As a result, the information is difficult to read, especially among patients with lower literacy skills [3]. One study found that only about half of the participants understood the information presented [4]. In another exploratory study, researchers used a mixed methods approach that combines eye-tracking technology, a survey, and qualitative data to explore self-reported measures of drug-risk reading and actual information recall. More than 80% of participants (n=29) claimed to have read or comprehended only half or more of the risk information. However, eye-tracking measures revealed limited to no understanding of risks and minimal unaided recall [5].

Consequently, patients cannot identify pivotal warnings from the guides, as evidenced by an increase in all medication-related hospitalizations by 117% from 1997 to 2008 [6]. Limited research in this area suggests that minimal patient engagement with information may contribute to unrecognized adverse effects that can lead to poor adherence. Furthermore, patient cognition and low literacy levels have a negative impact on medication adherence [7-9]. Therefore, the solution may not be whether or not the information was presented but rather *how* the information presented supports the cognition of the patient [10]. Moreover, understanding the patient’s cognitive processes could ultimately impact both intentional and unintentional medication nonadherence. Intentional nonadherence relates to stopping or altering prescribed medications through a perceived purpose, whereas unintentional medication nonadherence relates to specific circumstances that impede compliance, including forgetting to take a dose [11]. Both intentional and unintentional nonadherence lead to poor outcomes and uncontrolled disease states. Additionally, studies have detected a relationship between nonadherence and health literacy [12,13]. One study in particular investigated asthmatic patient perspectives of medication nonadherence to inhaled corticosteroids and observed that a factor for noncompliance was suboptimal knowledge of medications [14]. Moreover, health literacy is positively associated with cognition [15]. Thus, patients with lower health literacy and lower cognition have more serious adverse drug events [16]. By understanding cognition, novel solutions to medication nonadherence can potentially be developed. Previous studies in understanding cognitive processes mainly focused on the decision making of clinicians to support cognition [17-19]. Research on the aspects of understanding the patient’s cognition is still limited. Thus, a gap exists in the understanding of how patients manage complex medication information cognitively in the digital environment.

Currently, the patient’s cognition can be supported through effective medication information communication using digital platforms, such as smartphone apps. In particular, recent advancements in artificial intelligence (AI) embedded into smartphone apps have the potential for a significant impact to monitor and eventually comprehend different features of the patient's cognition [20,21].

We hypothesize that interactive information visualization using human factors design principles delivered through a smartphone platform has the potential to improve medication information retention. Infographics or interactive visualizations using pictures and illustrations have been effective for complex information communication and have proven the age-old saying “a picture is worth a thousand words” [22-25]. Furthermore, AI-based smartphone devices have proven to be effective in clinical trials and chronic disease management to improve medication adherence [21,26]. Utilizing advanced machine learning algorithms, smartphone apps have the potential to improve medication information delivery to patients. Specifically, algorithms based on AI can help advance our understanding of patients’ cognitive processes and provide solutions through digital health technology.

### Objective

Our goal is to improve the design of an innovative, AI-powered user interface based on human factors methodology to support the patient’s cognition, enhance the ability to comprehend medication information, and ultimately improve medication adherence. The aims are listed below:

1. Aim 1: identify barriers to medication information comprehension.
2. Aim 2: iteratively design and evaluate the AI-powered medication information visualization interface.
3. Aim 3: test the prototype for better comprehension and adherence to medication information.

## Methods

### Overview

We will use RxNorm and the Unified Medical Language System to design the AI infrastructure to unify medication and biomedical terminologies systematically. Generic medication names for the most prescribed and commonly used oral medications will be obtained from RxNorm. A recursive neural network (RNN)–based methodology was used for developing the AI-based algorithm. Artificial neural networks (ANNs) are modeled after the human brain and structured to recognize and recall information. An RNN is a type of ANN algorithm that operates on structured inputs of an acyclic graph. It is a type of architecture used in machine learning for various applications, such as predicting the weather, processing images, and language composition. We implemented this architecture and used deep learning algorithms using Amazon SageMaker for integrating the AI aspects into the smartphone app. We will use AI to monitor features of user analytics, use predictive tools to understand user interaction, and create a dynamic user environment of the app that changes based on the user’s previous input. The deep learning methodology of RNNs will guide us to develop such features. The user’s input will be reused to train the machine learning model.

### Aim 1: Identify Barriers to Medication Information Comprehension

#### Methods Overview

We will conduct a mixed methods study following these three steps:

1. Step 1: identify patients’ perceptions of fear and biases regarding medication information from a focus group study.
2. Step 2: the eye-tracking study will provide design configurations for the interface.
3. Step 3: psychological refractory period (PRP) will help us understand functionalities based on user profile variations.

#### Step 1: Observational Study

In the first phase, we will conduct multiple group interviews (ie, focus group methodology) for patients to interact to gain new knowledge and generate meaningful suggestions, opinions, and feedback [27]. Each focus group will have 5 patients. We will ask each patient to provide us with the name of a new medication, and we will print the Medication Guide per FDA to facilitate discussions. The study measures are (1) specific medication information on the leaflet that is hard to comprehend, (2) trigger points for information avoidance and perceived familiarity, and (3) patients’ suggestions. An initial coding framework will be developed by two coders to establish themes and subthemes from the transcript data. Refinement of coding will follow, and any disagreement between two coders will be resolved by consulting with a third coder.

#### Step 2: Eye-Tracking Study

In the second phase, we will design a web-based mock interface representing medication information in an interactive visualization format. We will use medication information from the FDA’s Medication Guides that are provided to patients for designing this mock-up. We will use the open-source software platform Pupil, a wearable, mobile, eye-tracking headset with one scene camera and one infrared-spectrum eye camera for dark pupil detection [28]. The video streams are read using Pupil capture software for real-time pupil detection, gaze mapping, recording, and other functions. We will use a mobile platform to display this information. Patients will be asked to wear the Pupil device and browse the web-based mock-up of medication information on a mobile device. They also will be asked to perform a series of tasks; for example, “find the most common side effect of the drug,” “find the storage instructions,” “find instructions on whether you can break the pill in half and take it,” etc. Patients will be asked to browse the interface data and provide overall feedback in a short semistructured verbal interview that will follow the task segment. The study measures are (1) specific areas in medication information where attention is focused, (2) real-time gaze movement using the Pupil software algorithm, and (3) accuracy, response time, and eye-movement data. For the data analysis, the Gaze History Length option for each surface decides how many recent gaze positions will be used to calculate the heat map based on gaze history. We will use annotation software to label timestamps of specific video gazing to understand the attention locus. Two researchers will assess the transcribed audio interviews to generate trigger points, user-centered design recommendations, and user preferences.

#### Step 3: Psychological Refractory Period Paradigm Study

In the third phase, we seek to understand users’ variations in their ability to complete two tasks in rapid succession for a goal-directed behavior; in our proposed research design, this understanding will be unpacked by determining the limits of dual-tasking by self-regulation and task-based measurement. The specific methodology for data collection is the PRP task, a tool provided by the National Institutes of Health Science of Behavior Change (SOBC) program [29-31]. The PRP gives specific instructions, which participants must follow as stated on the screen. The time, variation, and accuracy are measured through PRP and results are readily available to be downloaded after the session. The results will help us understand functionalities based on user profile variations, including age, health literacy, or any other physical and mental conditions. Patients will be given the test from the SOBC site from a web browser on a laptop computer. The study measures are (1) response time and (2) task-switching time. For the data analysis, we will note the variations of the response time based on patients’ ages and backgrounds to help create design features of buttons and clickable interface functional requirements in the design for the interface.

#### Study Subjects and Recruitment Methods

We will recruit 50 patients from our outpatient clinic sites. A research assistant will conduct a telephone interview for cognitive status to ensure potential participants meet the minimum cutoff score of 34 during cognitive screening based on TICS-M test for sound cognitive status and health literacy level [32]. Participants will come to our main site at a lab at the Western University of Health Sciences, Pomona, California. Inclusion criteria will be patients who are aged 18-65 years and have started at least one new medication to develop visualization information. The observational study (30 minutes), eye-tracking study (20 minutes), and PRP (10 minutes) will take around one hour, including instructions and filling out consent forms. Patients will be offered a US $100 gift card and access to a pharmacist consultation for the duration of the study.

#### Data Collection

We propose to collect the following data: (1) observation notes and audio recordings, (2) video data from Pupil on specific locations on gaze mapping, surface detection, and audio streaming data, (3) notes from user observations and audio of the short interviews after the eye-tracking study, and (4) task-switching and task-processing time from PRP.

#### Sample Size Justifications

Previous observation and eye-tracking studies used 10-90 participants and 5-40 participants, respectively [17,33,34]. We plan to recruit 50 patients for this aim.

#### Limitations

Patients may experience peer pressure to show conformity to medication information. We will have the moderator clarify to patients that the questions have no right or wrong answers. Some errors may result from gaze mapping, surface detection, and algorithm data. Therefore, we will calibrate the software and hardware before each study.

### Aim 2: Iteratively Design and Evaluate the AI-Powered, Medication Information Visualization Interface

#### Methods Overview

We will use an AI-based smartphone interface platform for our iterative design of the medication information interface. To develop such a platform, we will use the Amazon SageMaker engine for creating an AI-powered interface with our RNN-based algorithm. This interface will be able to take into account users’ interactions and translate the data into a data analytics–based back-end server. This prototype will be developed in Java and Python languages for both android and iOS platforms.

#### Human Factor–Based Iterative Design

We will use a human factor–based methodology to iteratively design the app from the functional requirements from Aim 1 using user-centered design principles [12,23,34]. We will use the usability inquiry approach for the iterative design to understand the users’ likes, dislikes, and needs [35,36]. The multidisciplinary research team, including 20 clinicians with diverse clinical backgrounds and 12 researchers, will then iteratively review and revise the app based on the written and verbal feedback related to the usability (ie, think-aloud methods), efficiency, and ease of use for 4 months or until no further revisions are identified. App usability will not be measured remotely. A research assistant will recruit clinicians and other team members for the iterative design process related to the front end of the prototype app. We will ask users to interact with the mock-up in the mobile interface and provide them with tasks using think-aloud methods. Think-aloud methods will provide rich verbal data about specific changes and functionalities of the initial mock-up [35,37]. We will audio record and screen record sessions, using Camtasia (TechSmith Corporation), to analyze verbal feedback from the think-aloud method and measure the mouse movements. We will analyze the data from the think-aloud sessions and screen recordings to identify design functionalities, and we will change the design based on feedback. The think-aloud sessions and screen recordings will provide us with specific user comments and interface design preferences. Our design team will incorporate those changes and take users’ feedback in an iterative process unless a final design agreement has been reached.

#### Contextual Medication Information for the Visualization

Two medication information expert pharmacists will develop contextual medication information—10 pieces of crucial information—that the patients should know [38]. This information may include indications for use, essential warnings, contraindications, storage information, typical side effects, and directions for use. We will use this information in the design of the visualization as well as use just the text version of the information as an intervention for Aim 3.

#### Usability Testing of the App

Two usability surveys will be emailed to users. The *first survey* instrument, which has been validated and created from the research on smartphone apps from the industry, is specifically designed for mobile interface design [39,40]. This Likert scale–based survey is based on a heuristic evaluation for easy, quick, and reliable assessment of the mobile user interface design [39]. The *second survey* instrument is the hierarchical task measure developed by the SOBC program. Hierarchical task analysis helps us to understand how users categorize tasks in hierarchical steps in their minds [41]. The hierarchical reinforcement learning task measures participants’ abilities to discover and use higher-order structures in their environment. Participants are presented with 18 stimuli composed of three dimensions: shape, orientation, and border color. The task requires that participants respond to stimuli by pressing one of three keys in response to each of the stimuli. In a “flat” condition, the keys are randomly associated with the shapes so that the participant must learn each association independently. We will iteratively change the design of the visualization based on the survey results.

#### Study Subjects and Recruitment Methods

We will recruit patients (18-65 years of age) at clinic sites during the discharge process with access to, and the ability to use, a smartphone device. A research assistant will conduct the modified Telephone Interview for Cognitive Status (TICS-M) test for cognitive status to ensure potential participants meet the inclusion criteria of a minimum cutoff score of 34 for sound cognitive status and health literacy level [32]. Patients will be given a US $50 gift card for participation. We will load our medication information tool remotely into the users’ app and send out an e-survey for ratings and feedback. The data from the usability surveys will be sent to a HIPPA (Health Insurance Portability and Accountability Act)-protected secured server.

#### Data Analysis

For the hierarchical task, the results will be shown in graphs with the percentage of error of participants’ abilities to simplify tasks. We will correlate the means of the graphs with the results from the survey instruments for a personalized design.

#### Sample Size Justifications

Previous studies have included usability surveys with 96-404 participants [40,41]. We plan to recruit 300 patients from our outpatient clinics at the Western University of Health Sciences.

#### Limitations

We may receive numerous users’ input about the interface. The research team will apply only the changes that correspond to the current literature of design conforming to patient safety [42].

### Aim 3: Test the Prototype for Better Comprehension and Adherence to Medication Information

Our hypothesis for this aim is that the AI-powered visualization tool in the app improves medication information comprehension and adherence when compared with written medication information and contextual plain-text information.

#### Study Design

**Figure 1:**
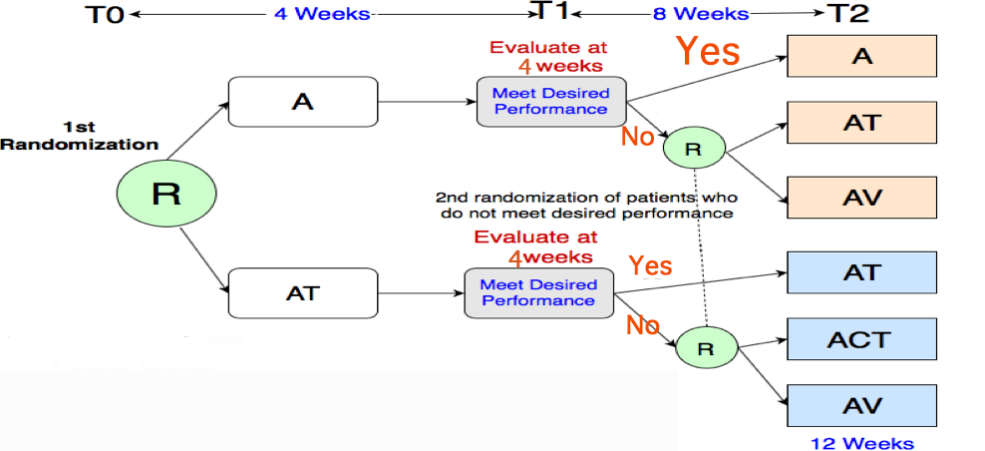
Study design. The participants are randomized first at R: one group uses only the app (A) and another group is given the app with medication information text (AT). At time point 1 (T1), after 4 weeks, both groups are evaluated for information recall (>80%). Then, after a second randomization (R), the participants are divided into the following groups: app with medication information text (AT), app with contextual medication information text (ACT), and app with visualization (AV). T0: baseline; T2: time point 2 (after 12 weeks).

The study has a single-blind and sequentially randomized design. The full design of the study will include two arms with 2 (modality: app versus app with text) × 2 (format: an app with visualization versus an app with contextual medication text information) × 2 (context: contextual medication information versus information without context) + 1 (control: app) design. The design is depicted in Figure 1. The randomized trial using binary options (*yes* or *no*) provides the stratification needed to identify groups that are prone to get benefits from the different presentations of medication information. The results from the sequential randomized design will help us detect statistically significant differences using a larger sample size. The first randomization helps with filtering patients who have better information recall regardless of the app. The second randomization helps with finding the actual effects of the tool when compared with different interventions.

#### Intervention

The study will include three intervention conditions loaded into the smartphone app platform. We can better compare the effects of the medication information visualization tool by comparing the outcomes of these three interventions to understand the impacts of our visualization. In the first condition, the app with medication information text (AT), the medication text information will be loaded into the app platform. This medication text is the Medication Guide, which is approved by the FDA. In the second condition, the app with contextual medication information text (ACT) is shown as a printed-text version. This contextual information (ie, 10 pieces of crucial information patients should know) that we included in our visualization will be shown only as the text version in this intervention. In the third condition, an app with visualization (AV), the interactive app will be loaded with our medication information visualization tool, including the visual format of the contextual information.

#### Study Subjects and Recruitment Methods

We will recruit patients (18-65 years of age) from our clinics. Inclusion criteria will include participants who have access to a smartphone, can provide their consent, do not have any cognitive impairment, and who have at least two medications on profile for which we have created a visualization. At the time of recruitment, we will ensure that the patients meet the minimum cutoff score of 34 during cognitive screening using the TICS-M test and health literacy level [32]. Eligible patients will be allocated in a 1:1 ratio to the two arms of the study according to a computer-generated random sequence stratified by third-party software.

#### Procedure

In the first randomization, patients will be randomized into two groups at baseline. The control group (ie, the app-only [A] group) will have the smartphone app with no visualization, and the intervention group will have the app with text information only as in the current Medication Guide (AT) [43]. At 4 weeks, patients from both the control and intervention groups from both arms, who have scores of 80% or higher in the information recall survey, will continue in the study with the same intervention, in either the A or AT group. Those who do not meet the performance cutoff will be rerandomized further into two groups for each arm. In the second randomization at 4 weeks, the control group from the first arm (A) will have the smartphone app with just text information about medication (AT), and the intervention group will have the smartphone app with the prototype of medication information visualization (AV). The control group from the second arm (AT) will have the app with contextual medication information in a text format (ACT), and the intervention group will have the app with our visualization design (AV). This part of the study will continue for 8 weeks. The monitoring is not a continuous process. Only the principal investigator will have access to the main file and will check the adherence and patient analytics at 4 and 8 weeks.

#### Survey Tools

Three survey measures will be used. The first survey will be used to understand information recall; we will design a timed questionnaire, with *true* or *false* responses, for each medication in a patient’s regimen that includes the 10 pieces of crucial medication information we embedded into the visualization interface. Each patient will be tested on different medications at 4 and 12 weeks to show understanding and knowledge. For example, if a patient takes simvastatin, metformin, and losartan, only those specific medication-related questions will be asked. The second survey will help us to understand the patient’s perception of medication knowledge. We will use a validated survey instrument to assess patients’ attitudes, confidence, and perceived medication knowledge [44]. This instrument was validated with high internal consistency (Cronbach α=.833). The third survey will use the 8-item Morisky Medication Adherence Scale (MMAS-8). The MMAS-8, whose scores range from 0 to 8 with lower scores indicating lower adherence, is a widely used tool for self-reported medication adherence; the scale was found to be reliable and significantly associated with adherence control (*P*<.05), with 93% sensitivity as well as 53% specificity for low adherence in a validation study [37].

#### Data Collection

We will collect demographics data at baseline. Data will be collected at 4 and 12 weeks. The 12-week time point will be considered the primary endpoint for the study. The AI platform on the smartphone app’s back end will provide patient analytics on the usage of the mobile app (ie, intake of medications in the app diary and access frequencies).

#### Study Measures

The following study measures will be conducted:

1. Medication information comprehension from the first survey (primary outcome) [45]; the comprehension measure will be a composite of three subscales:
  - Relevance to understanding patients’ attitudes,
  - Patients’ levels of confidence and knowledge of medication use, measured using a Likert scale, and
  - Information recall subscale, measured using a dichotomous scale (ie, true or false).
2. Survey on patients’ perceptions of medication information.
3. Medication adherence (secondary outcome) will be operationalized by the following:
  - MMAS-8 [46] survey score; the dichotomous response categories are yes or no for each item, and
  - Analytics of the patients’ daily intake of medications diary from the smartphone app.

#### Data Analysis

Demographic information will be presented using descriptive statistics. We will analyze the groups using *t* tests to detect differences between the means for continuous data. Chi-square analysis will be used to evaluate differences between arms for primary and secondary outcomes. We will use the univariate analysis of variance to calculate the mean differences between groups. If the distribution is not normal, we will use general linear modeling for the data analysis [47,48]. The AI-based analytics data from the smartphone will show us the patients' medication intake and we will correlate that score with our interventions.

#### Sample Size Justifications

To detect a 1-point improvement on the Likert scale and 80% power at the 5% significance level, the study would require 100 subjects in each group with 1:1 allocation for the second randomization (ie, AT: AV and ACT: AV). To allow for an 11% loss due to dropouts and those lost to follow-up, we would need to recruit 888 patients, allowing up to 50% of the patients to move to the second randomization following the first randomization with a score of 80% or higher in the medication recall questionnaire survey with assigned intervention. Recruitment of 888 patients is also sufficient to detect a 1-point improvement on the MMAS-8 (the secondary outcome), with 80% power at 5% significance. Therefore, our target is to recruit 900 patients.

#### Limitations

We expect to enroll a large number of patients for this sequential randomized design. We do not anticipate significant problems because our clinics are excellent sites for recruitment. The recruiting advantage is due to the academic affiliations with faculty and residents. However, given our large sample size requirements, to the extent that we cannot recruit around 888 patients, our study will lack rigor.

## Results

We will conduct preliminary data collection for the studies in 2021. Results are expected to be published in 2022.

## Discussion

### Overview

This protocol addresses the problem of creating an AI-powered smartphone app to engage and predict user analytics. This unique idea builds upon an understanding of how users interact with complex medication task information and interactively visualize crucial medication information that frequently is ignored. Previous studies have also successfully shown that informatics platforms have the potential to empower patients [49]. While AI-based algorithms have been used in predicting health outcomes [20,50], very few studies have looked into the user's interaction analytics and ways to build better visualization techniques to engage the user [51-54]. This protocol focuses on creating uniform data standards that are crucial for efficient health information exchange [55]. This protocol provides future researchers and visualization designers with a new and innovative way to design and improve health care smartphone apps.

### Conclusions

This study will lead the future of innovative AI-powered smartphone app design and act as the aid to improve medication risk comprehension, which may ultimately improve medication adherence. The results from this study also open up future research opportunities to understand how patients manage complex medication information and will inform the format and design for innovative AI-powered digital interfaces for Medication Guides.

## Abbreviations

A: app only
ACT: app with contextual medication information text
AI: artificial intelligence
ANN: artificial neural network
AT: app with medication information text
AV: app with visualization
FDA: US Food and Drug Administration
HIPPA: Health Insurance Portability and Accountability Act
MMAS-8: 8-item Morisky Medication Adherence Scale
PRP: psychological refractory period
RNN: recursive neural network
SOBC: Science of Behavior Change
TICS-M: modified Telephone Interview for Cognitive Status
